# The Physiological Mechanisms Behind the Earlywood-To-Latewood Transition: A Process-Based Modeling Approach

**DOI:** 10.3389/fpls.2018.01053

**Published:** 2018-07-20

**Authors:** Fabrizio Cartenì, Annie Deslauriers, Sergio Rossi, Hubert Morin, Veronica De Micco, Stefano Mazzoleni, Francesco Giannino

**Affiliations:** ^1^Department of Agricultural Sciences, University of Naples Federico II, Portici, Italy; ^2^Département des Sciences Fondamentales, Université du Québec à Chicoutimi, Chicoutimi, QC, Canada; ^3^Key Laboratory of Vegetation Restoration and Management of Degraded Ecosystems, Guangdong Provincial Key Laboratory of Applied Botany, Chinese Academy of Sciences, Guangzhou, China

**Keywords:** carbon allocation, cell enlargement, cell-wall thickening, sugar availability, tree ring, wood anatomy, xylogenesis

## Abstract

In extratropical ecosystems, the growth of trees is cyclic, producing tree rings composed of large-lumen and thin-walled cells (earlywood) alternating with narrow-lumen and thick-walled cells (latewood). So far, the physiology behind wood formation processes and the associated kinetics has rarely been considered to explain this pattern. We developed a process-based mechanistic model that simulates the development of conifer tracheids, explicitly considering the processes of cell enlargement and the deposition and lignification of cell walls. The model assumes that (1) wall deposition gradually slows down cell enlargement and (2) the deposition of cellulose and lignin is regulated by the availability of soluble sugars. The model reliably reproduces the anatomical traits and kinetics of the tracheids of four conifer species. At the beginning of the growing season, low sugar availability in the cambium results in slow wall deposition that allows for a longer enlargement time; thus, large cells with thin walls (i.e., earlywood) are produced. In late summer and early autumn, high sugar availability produces narrower cells having thick cell walls (i.e., latewood). This modeling framework provides a mechanistic link between plant ecophysiology and wood phenology and significantly contributes to understanding the role of sugar availability during xylogenesis.

## Introduction

Current research on the dynamics of tree-ring formation in conifers has provided new insights into how the rate and duration of growth processes (i.e., xylem-cell production, enlargement and wall formation) control the size of the xylem conduits both across the tree-ring width (Cuny et al., 2013, 2014; Balducci et al., 2016) and along the stem hydraulic pathway (Anfodillo et al., 2012). These processes define the anatomical structure of the tree ring and determine the efficiency and safety of water transport, the storage capacity of water and reserves, and the mechanical resistance of the stem (Chave et al., 2009; Fonti et al., 2010). In extratropical ecosystems, across the tree ring, which is produced from spring to autumn, a decrease in tracheid lumen size is observed associated to a progressive reduction in the duration of cell enlargement, while cell-wall thickness increases along with the time spent completing cell-wall deposition and lignification (Wodzicki, 1971; Deslauriers et al., 2003; Rossi et al., 2006b; Cuny and Rathgeber, 2016). However, as already pointed out by Vaganov et al. (2011), the physiological factors influencing the rate and duration of the tracheid formation processes that determine their specific morphology, remain poorly understood.

According to Sorce et al. (2013), the physiological processes of tracheid differentiation depend on various factors, including resource availability (e.g., water, carbon and nutrients), growth regulators (i.e., hormones) and environmental factors (e.g., temperature, photoperiod). After cambial cell division, a growing cell requires water, free sugars and amino acids to build and maintain the turgor pressure for its expansion (Koch, 2004; Pantin et al., 2012; Deslauriers et al., 2014; Steppe et al., 2015), sucrose to build up secondary cell wall (Uggla et al., 2001; Simard et al., 2013; Deslauriers et al., 2016) and time to complete the maturation processes (Deslauriers et al., 2003; Schiestl-Aalto et al., 2015).

Once the activity of primary and secondary meristems begins in the early spring, sink competition for carbon allocation is unavoidable. In conifers, the resumption of growth activity in the cambium (in the stem) and buds (in the canopy) occurs at the same time (Antonucci et al., 2015). Thus, to start cambial activity and develop the first tracheids, the nearby starch reserves are used (Hansen and Beck, 1990; Oribe et al., 2003; Begum et al., 2013), while reserves from twigs and newly synthetized photosynthates—provided by older needles—are used to supply the developing buds (Hansen and Beck, 1994; Heinrich et al., 2015). Heinrich et al. (2015) investigated C partitioning of freshly assimilated C into tree compartments following in situ ^13^C allocation 15 days after bud break and they observed that it mainly reflects the high metabolic C demand of the canopy (85% of the allocated ^13^C) with only a minor proportion (1.6%) translocated to the stem. At the same time, the first earlywood tracheids in the stem are either completing differentiation or mature (Huang et al., 2014). Therefore, despite being an active sink, the xylogenesis process in the stem receives a minor portion of the recently assimilated C when shoots and needles in the canopy are actively growing. Accordingly, as already proposed by previous researchers (Richardson and Dinwoodie, 1960; Larson, 1964; Gordon and Larson, 1968), primary growth in the tree canopy could influence the sugar availability for xylogenesis and contribute to the anatomical changes from earlywood to latewood. The general anatomical pattern observed in conifers, where each tree ring is composed of large and thin-walled earlywood tracheids followed by narrow and thick-walled latewood tracheids, could thus be determined by the C allocation pattern during the growing season.

The role of indole-3-acetic acid (IAA) on xylogenesis has also been extensively investigated. Early research on the subject reported that exogenous applications of IAA to cambia that were forming latewood tracheids could induce the formation of earlywood tracheids (Larson, 1960). This evidence led to the conclusion that a reduction in the concentration of IAA could be responsible for the transition from earlywood to latewood although subsequent studies provided conflicting results. Some evidence suggests that IAA, produced in young leaves and transported downwards through the stem, could be directly responsible for both radial (within the tree ring) and longitudinal (along the stem) patterns of tracheids traits (reviewed in Aloni, 2015). However, heating the trunk before the beginning of the growing season reactivated the cambial region and the production of layers of earlywood cells in several conifer species (e.g., Gričar et al., 2006, 2007; Begum et al., 2012) at a time where IAA levels should have been comparable to those associated to the formation of latewood cells. The work of Uggla et al. (2001) clearly showed that the earlywood to latewood transition and the cessation of cell division were not associated with a decreasing concentration of auxin in cambial cells. On the other hand, the authors observed a clear reduction in the width of the auxin gradient, which has a peak in the cambial cells and steeply decreases toward the developing xylem. They argued that this could provide a spatial cue for the transition from the expansion to the thickening phase. Very recently, a theoretical model capturing the spatial-temporal interactions between meristematic cells and morphogenetic signals was proposed by Hartmann et al. (2017). The authors demonstrated how a single morphogenetic gradient (e.g., IAA) could explain xylem radial growth and tissue zonation but failed to explain final cell sizes observed in tree rings. They speculated that a second gradient might be at work to explain both emergent properties.

Several mathematical models have been developed to simulate wood formation in conifers, each model having different assumptions. In particular, climatic and physiological factors were included in various models of xylogenesis, either with validation by tree-ring anatomical data (Fritts et al., 1999; Vaganov et al., 2011; Drew and Downes, 2015; Schiestl-Aalto et al., 2015; Li et al., 2017) or without (Hölttä et al., 2010; Brüggemann et al., 2011; Hartmann et al., 2017). Three of these models (Hölttä et al., 2010; Drew and Downes, 2015; Schiestl-Aalto et al., 2015) highlighted the importance of including sugar and water availability as valuable factors in the growth kinetics. In Drew and Downes (2015), the tracheid morphology across the tree-ring width was driven by changes in non-structural carbohydrates and water availability, while the transition from earlywood to latewood was directly controlled by photoperiod. As day length increased, carbon allocation was preferentially directed toward cell division and enlargement, while the process of cell-wall thickening was prioritized after the summer solstice. Schiestl-Aalto et al. (2015) based cell production on the thermal time concept and sink-source relationships. Finally, the Vaganov–Shashkin model (Vaganov et al., 2006, 2011) relied on external environmental conditions where the daily growth rate was calculated based on a number of environmental factors used to estimate the kinetics of wood formation and the anatomical features of tree rings.

Although providing reliable predictions, the above-mentioned models assumed fixed *ad hoc* rules for the succession of cell-differentiation phases. Furthermore, none explicitly considered possible feedback between the enlargement processes and deposition of new wall material (i.e., downregulation Huang et al., 2012), which could have a significant impact on the emergent properties of the system, i.e., on the variations in cell traits observed across tree rings.

In this work, we present a process-based model to explain the observed intra-annual variations of the traits of xylem cells by explicitly simulating cell enlargement as well as cell-wall deposition and lignification. With this model—assuming that sugar availability for cambial activity increases when primary growth ends—we aim specifically to understand how such seasonal changes could impact the general anatomical pattern of tracheids across the tree ring and the rate and duration of cell enlargement and cell-wall formation.

## Methods

### Model description and mathematical formulation

For simplicity, the xylogenesis process is often described in five steps: (1) the periclinal division of a cambial mother cell into two daughter cells; (2) the increase in volume of the daughter cells combined with integration of new polymers into the walls; (3) the formation of different layers of secondary wall due to the deposition of cellulose and non-cellulosic polysaccharides; (4) cell-wall lignification; and (5) programmed cell death (Plomion et al., 2001). During the increase in volume (commonly termed cell enlargement), wall loosening enables growth in response to increased turgor pressure after water uptake (Cosgrove, 2005; Proseus and Boyer, 2005). The deposition of cellulose microfibrils then prevents the growing cell from breaking, while various components, such as hemicellulose and pectins, act as cross links and load-bearing elements between microfibrils. However, as cell enlargement continues over days, increased Ca^2+^-pectate complexes reduces cell-wall extensibility (Peaucelle et al., 2012). This downregulates growth due to the progressive thickening and stiffening of the cell wall (Huang et al., 2012).

Here we present a novel process-based model that reproduces the morphogenetic development of differentiating xylem tracheids, i.e., the phases of cell enlargement, cell-wall deposition and lignification. The initial state of the simulated cell is that of a derivative produced by the cambium toward the xylem side and enclosed by a layer of primary cell wall. The cell is described in transverse section by three state variables that represent the entire cell area (*CA*, μm^2^), the area covered by the wall (*WA*, μm^2^) and the portion of lignified wall (*LWA*, μm^2^).

The model is based on the assumptions that (1) each cell grows independently of the others; (2) cells are rectangular in cross-section and enlarge only radially; (3) cell death occurs when the wall is completely lignified (Mittler and Lam, 1995); (4) cell enlargement stops when a threshold thickness is reached (Huang et al., 2012); (5) in the cell, sugar availability changes during the growing season but remains constant during the development of a single cell through the enlargement and secondary wall-thickening phases after the cambial division (Uggla et al., 2001); and (6) the wall formation rate, i.e., microfibril deposition and lignification, depends on both concentration of available sugars and length of the lumen perimeter (Verbančič et al., 2017). Indeed, while cellulose microfibrils are synthesized through the hexameric complexes directly in the plasma membrane toward the developing cell wall, other compounds (e.g., monolignols) are synthesized in the protoplast and are transported to the wall surface by vesicles whose trafficking is quicker and more efficient in cells having a larger contact surface between the wall and lumen.

Following the principle of parsimony, the effect of environmental factors (i.e., temperature and water availability) on the considered processes was not explicitly included in the model. In particular, water availability was assumed to not be a limiting factor, while temperature has a direct effect only on the wall deposition of the last latewood cells (Cuny and Rathgeber, 2016).

According to these assumptions, the dynamics of cell enlargement are described by the differential equation:

(1) $$\begin{array}{l} \frac{dCA}{dt}=v_{c}\;\cdot \;CA\;\cdot \;\left(1-\frac{CA}{CA_{\max}}\right)\;\cdot \;\left(1-\min\left(1,\frac{WT}{WT^{*}}\right)\right) \end{array}$$

where *v*_*c*_ is the specific cell enlargement rate, *CA*_*max*_ is maximum surface area that the cell can attain, *WT* is the cell wall thickness, and WT^*^ is the threshold thickness at which the cell enlargement process stops. The most important feature of Equation (1) is its dependency on wall thickness [1-min(1,WT/WT^*^)], which is derived directly from the fourth assumption. The remainder of the equation represents a simple logistic growth in which the term *CA*_*max*_ is assumed to be the theoretical upper limit of cell expansion in the absence of wall deposition. Then, based on the assumption of a rectangular shape of the cell, cell-wall thickness is defined by:

(2) $$\begin{array}{l} WT=\frac{2\left(CTD+CRD\right)-\sqrt{4{\left(CTD+CRD\right)}^{2}-16WA}}{8} \end{array}$$

where *CTD* and *CRD* are the cell tangential and cell radial dimensions, respectively. As no tangential growth was assumed in the model, *CTD* is constant during morphogenesis, while *CRD* is calculated at each time step (*CRD* = *CA/CTD*).

The dynamics of cell-wall deposition are described by the differential equation:

(3) $$\begin{array}{l} \frac{dWA}{dt}=v_{w}\cdot S_{i}\cdot \left(1-\frac{WA}{WA_{\max}}\right)\cdot \left(1-\frac{1}{{\left(1+\frac{CA-WA}{m_{w}}\right)}^{s_{w}}}\right)\cdot Death \end{array}$$

where *v*_*w*_ is the specific rate of wall deposition, *S*_*i*_ is sugar availability within the *i*^*th*^ cell of the ring, *WA*_*max*_ is the maximum amount of wall that a cell can accumulate and *m*_*w*_ and *s*_*w*_ are calibration parameters. *Death* is a Boolean variable that sets the death of the cell when the wall is completely lignified according to:

(4) $$\begin{array}{l} Death=\{\begin{array}{c} 0,\;LWA\geq WA\\ 1,\;LWA<WA \end{array} \end{array}$$

The dynamics of cell-wall lignification are described through the differential equation:

(5) $$\begin{array}{l} \frac{dLWA}{dt}=v_{l}\cdot S_{i}\cdot \left(1-\frac{1}{{\left(1+\frac{CA-WA}{m_{l}}\right)}^{s_{l}}}\right)\cdot Death \end{array}$$

where *v*_*l*_ is the specific wall lignification rate, while *m*_*l*_ and *s*_*l*_ are both calibration parameters (Table 1).

**Table 1:** Explanation and simulation values of the symbols used in the model.

	**Symbol**	**Description**	***Pinus cembra***	***Picea abies***	***Larix decidua***	***Picea mariana***	**Units**
**Fixed**	*CA_0_*	Cell area initial value	327	248	308	362	μm^2^
	*WA_0_*	Wall area initial value	41	35	39	43	μm^2^
	*LWA_0_*	Lignified area initial value	0	0	0	0	μm^2^
	*CTD*	Cell tangential diameter	31	27	30	33	μm
	*WT_0_*	Wall thickness initial value	0.5	0.5	0.5	0.5	μm
**Calibrated**	*v_*c*_*	Enlargement max rate	0.39	0.44	0.39	0.41	d^−1^
	*CA_*max*_*	Max cell area	3180	2206	2989	1402	μm^2^
	WT^*^	Wall thickness threshold	1.10	1.02	1.85	1.71	μm
	*v_*w*_*	Wall deposition max rate	8.54	8.18	11.26	11.43	μm^2^ d^−1^
	*WA_*max*_*	Max wall surface	915	1145	553	596	μm^2^
	*m_*w*_*	Cellulose deposition rate calibration parameter	505	503	124	193	μm
	*s_*w*_*	Cellulose deposition rate calibration parameter	5.58	5.34	2.77	4.18	–
	*v_*l*_*	Lignin deposition max rate	7.79	8.23	10.77	12.96	μm^2^ d^−1^
	*m_*l*_*	Lignin deposition rate calibration parameter	515	523	108	100	μm
	*s_*l*_*	Lignin deposition rate calibration parameter	5.63	5.44	0.56	0.45	–
	*a*	Sugar curve calibration parameter	1.77	1.46	2.78	0.03	–
	*b*	Sugar curve calibration parameter	−0.07	−0.10	−0.53	4.15	–
	*c*	Sugar curve calibration parameter	1.60	1.81	1.83	0.79	–

**Figure 1:**
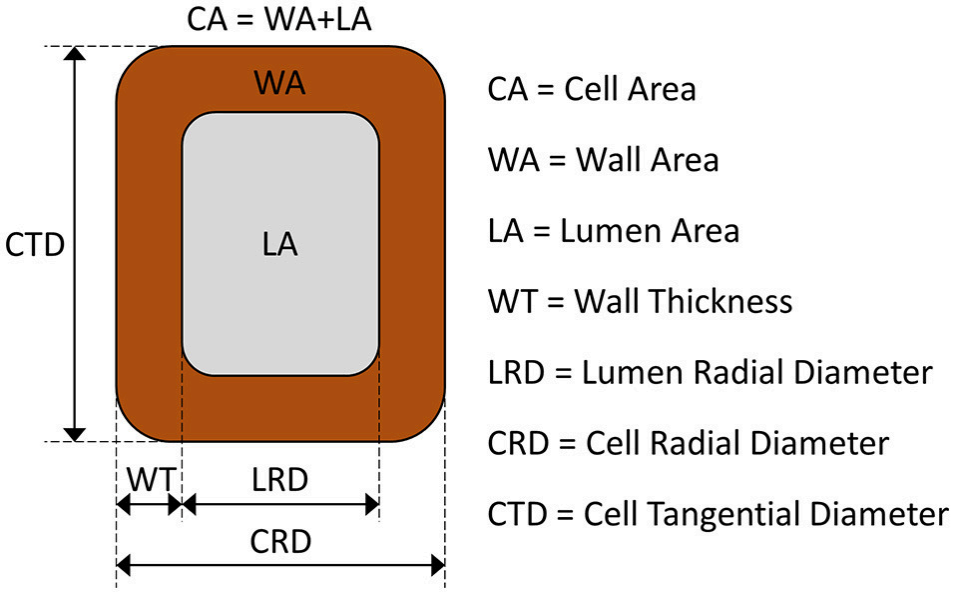
Representation of tracheid dimensions and abbreviations.

The measured values of the lumen area (*LA*), lumen radial diameter (*LRD*) and wall thickness (*WT*) were used to derive the cell tangential diameter (*CTD* = *LA/LRD* + *2WT*). From observations of the derivatives produced by the cambium of several conifer species, we measured the initial cell radial diameter (*CRD*_0_) and calculated the initial value of the state variable *CA* (*CA*_0_ = *CTD* × *CRD*_0_). The initial value of the cell-wall area (*WA*_0_), namely the area of the primary wall, was calculated as:

(6) $$\begin{array}{l} WA_{0}\;=\;2CTD\cdot WT_{0}+2WT_{0}\frac{CA_{0}}{CTD-2WT_{0}} \end{array}$$

where *WT*_0_ is the primary wall thickness. All symbols with their descriptions, values and units are summarized in Table 1. A schematic representation of a tracheid and its dimensions is depicted in Figure 1.

### Data collection

Data were collected from permanent plots in Italy in 2001 and in Canada from 1999–2004. Fifteen trees of *Larix decidua, Pinus cembra* and *Picea abies* (five trees per species) were selected at tree line in the eastern Italian Alps (46°27′ N, 12°08′ E, 2080 m a.s.l.). Twenty trees of *Picea mariana* were selected in the boreal forest of Quebec (48°13′ N, 71°15′ W, 338 m a.s.l.). All trees were adult, dominant or co-dominant individuals, having healthy crown and upright stems and lacking reaction wood.

Wood microcores (2.4 mm in diameter) were collected in autumn at a height of 1.3 m on the stems using an increment puncher or Trephor (Rossi et al., 2006a). The microcores were dehydrated through successive immersions in ethanol and D-limonene and then embedded in paraffin. Transverse sections, 6–10-μm-thick, were cut using a rotary microtome (Rossi et al., 2006a). The sections were stained with safranin (1% in water) and fixed with Eukitt. A camera mounted on an optical microscope recorded numerical images at magnifications of 250–400×. Cell features—lumen area (LA), LRD and wall thickness (WT)—were measured on three radial rows per section using WinCELL (Regent Instruments Inc., Canada) by producing tracheidograms, ordered series of variation in tracheid dimension along the radius (Deslauriers et al., 2003). All tracheidograms were standardized to the mean number of cells produced by each species (Vaganov, 1990) and the data were averaged for each cell to obtain mean tracheidograms that represent the general tree-ring structure of a species. Tracheids were then classified as belonging to earlywood or latewood according to Mork's formula, which classifies all cells having lumen 2 × smaller than a double wall as latewood (Denne, 1989).

### Model calibration and numerical simulations

The model was first developed in the SIMILE (Simulistics Ltd.) visual modeling environment to facilitate discussion within the multidisciplinary team during the implementation phases. The mathematical equations were then integrated using MATLAB R2016b (the MathWorks) with a non-adaptive solver that implemented the classical Runge-Kutta method of Order 4. The equations were solved separately for each cell across the tree ring, and the final outputs were then aggregated and compared with the measured tracheidograms dataset. Based on the initial assumptions, the parameter *S* (i.e., the concentration of available sugars in the cell) was assumed to be constant within each run of a single cell, but it could vary between the different cells of the ring. Two functions of sugar variation across the ring were tested, an exponential and a quadratic curve using the following formulas:

$$\begin{array}{l} S_{i}=ax_{i}^{2}+bx_{i}+c;\\ S_{i}=ae^{bx_{i}}+c; \end{array}$$

where *a, b* and *c* are the calibration parameters and *x*_*i*_ is the relative position of the *i*^*th*^ cell within the ring, which assumes values ranging from 0 (first cell) to 1 (last cell).

Model calibration was performed by minimizing the sum of the squared errors (SSE) according to:

$$\begin{array}{l} SSE={\sum_{i=1}^{n_{1}}\left(WT_{i}-WT_{i}^{*}\right)}^{2}+{\sum_{i=1}^{n_{2}}\left(LRD_{i}-LRD_{i}^{*}\right)}^{2}\\ \;+{\sum_{i=1}^{n_{3}}\left(LA_{i}-LA_{i}^{*}\right)}^{2}, \end{array}$$

where *n*_1_, *n*_2_ and *n*_3_ are the number of samples per observed output, *WT*_*i*_, *LRD*_*i*_, and *LA*_*i*_ are the values of the *i*^*th*^ measured outputs, and $WT_{i}^{*}$, $LRD_{i}^{*}$ and $LA_{i}^{*}$ are the values of the *i*^*th*^ outputs predicted by the model. The minimization was performed using the *fminsearch* MATLAB routine that implements a Nelder–Mead simplex algorithm (Lagarias et al., 1998). The list of calibrated parameters is reported in Table 1.

## Results

### Temporal dynamics of a tracheid development

**Figure 2:**
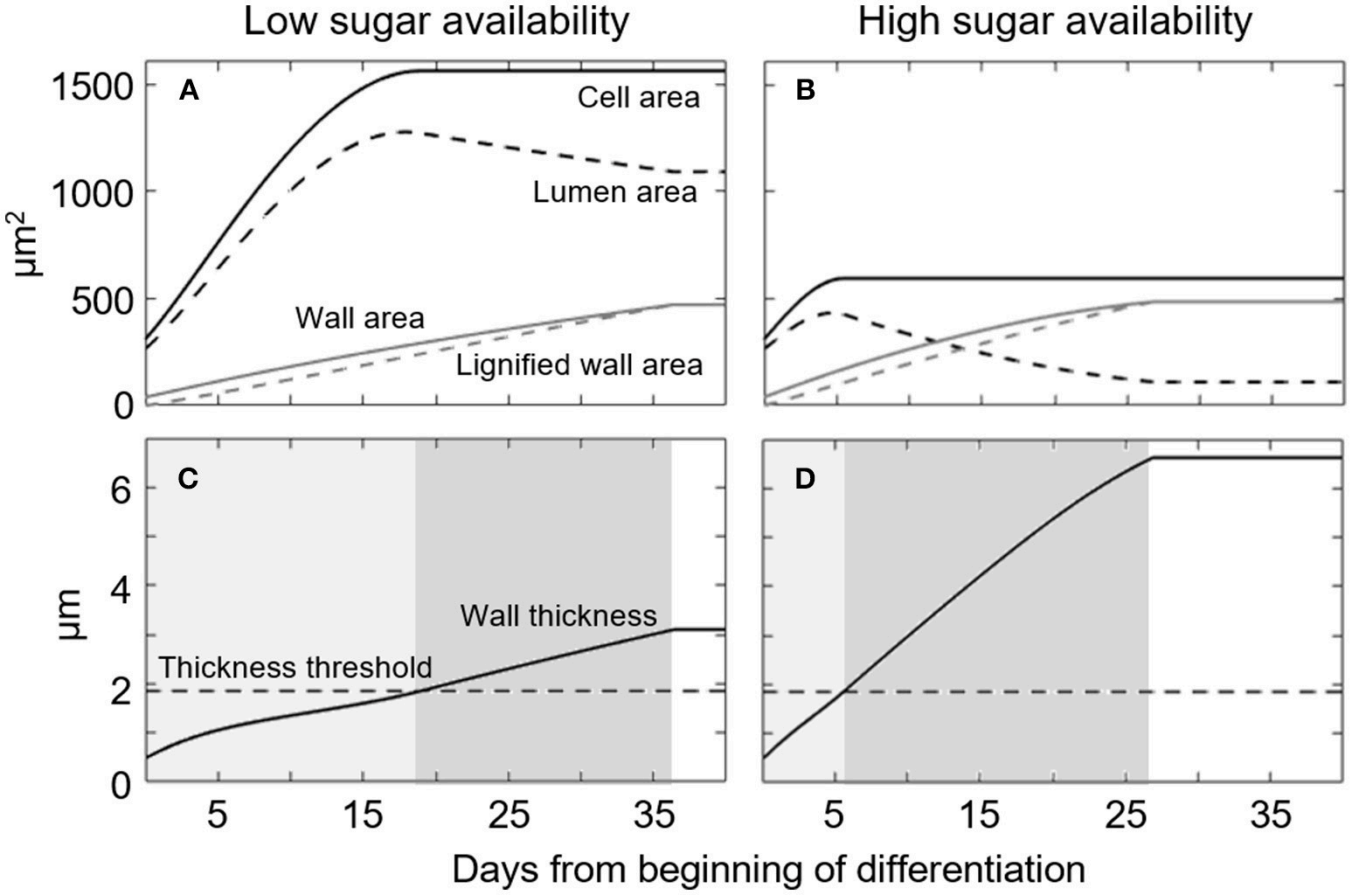
Simulated temporal dynamics of a single cell of *Larix decidua* exposed to either low (*S* = 1.58) or high (*S* = 2.65) sugar availability. **(A,B)** Temporal dynamics of simulated Cell area, Lumen area, Wall area and Lignified wall area. **(C,D)** Temporal dynamic of simulated Wall thickness. Light and dark gray areas represent phases of cell enlargement and wall thickening, respectively. Simulation parameters are reported in Table 1.

To understand model behavior, we performed a simulation of the temporal dynamics of cell formation for *L. decidua* at two markedly diverging concentrations of available sugars (Figure 2). These two model runs simulated the main tracheid formation variables over time (i.e., cell, lumen, and wall area). For both sugar concentrations, the initial growth phase is characterized by a rapid expansion of the cell and lumen areas (Figures 2A,B). Cell-wall area (Figures 2A,B) and thickness (Figures 2C,D) increase steadily following a linear trend. Consistent with the model's assumptions, the wall-thickening process slows the cell area expansion, finally causing expansion to stop when the thickness reaches the threshold value WT^*^; thus, we can determine the total tracheid area and duration of the expansion phase (Figures 2C,D, light gray areas). However, while the area reaches an upper asymptote at the end of cell expansion, the LA begins to decrease as the deposition of the cell wall increases. This reduction in LA produces a negative feedback on the wall deposition process, which slows down progressively. A reduced wall deposition rate allows for the wall lignification process to be completed. This results in cell death and the end of the wall-thickening phase (Figures 2C,D, dark gray areas).

The same developmental sequence occurs under low and high sugar availability (Figures 2A–D respectively), but with important differences in terms of the rate and duration of the different phases. According to the model, low sugar availability results in a slow cell-wall deposition and thus an extended period available for cell expansion. This occurs as the wall thickness threshold (WT^*^) is reached slowly (18 days), leaving a long time for the cell area to expand before reaching its asymptote, thereby producing a large cell (Figures 2A,C). The larger cell area, combined with a slower wall deposition rate, reduces the overall wall thickness and duration of the process (ca. 18 days, Figure 2C, dark gray area). On the contrary, only 6 days were necessary to reach WT^*^ when there was a high sugar availability, leaving a much shorter time for cell expansion. Finally, the smaller cell area and the thicker wall formed under conditions of high sugar availability increases the duration of lignin deposition across the cell wall; this lasted ca. 21 days (Figure 2D, dark gray area).

### Cell traits across the tree ring

**Figure 3:**
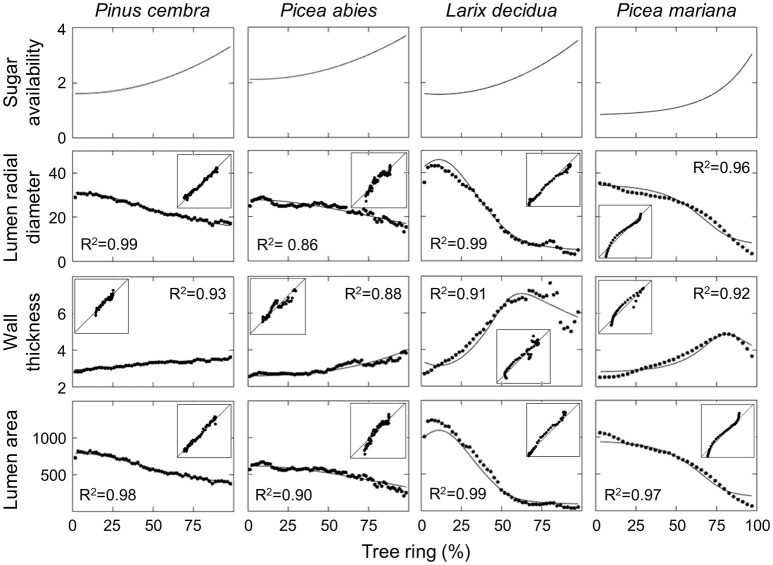
Simulated and observed anatomical features of tracheids across the tree ring for different conifer species. Insets represent the correlation plots between observations (y-axis) and simulations (x-axis). Model parameters and the initial values of state variables are reported in Table 1.

For all considered species (except for *P. mariana*), a best-fit between the simulations and measurements of LA, LRD and WT was obtained using a quadratic function of sugar availability. An exponential curve was best for *P. mariana* (Figure 3, first row). Both *P. cembra* and *P. abies* had very similar anatomical features, with a constant decrease in LRD and LA values and an increase in WT values across the tree ring (Figure 3, first two columns). LRD decreased from 30.8 to 16.3 μm in *P. cembra* and from 28.7 to 13.0 μm in *P. abies*. Consistent with LRD, LA was also slightly higher in *P. cembra* (between 819.9 and 373.1 μm^2^) than in *P. abies* (between 665.2 and 203.9 μm^2^), while WT ranged between 2.8 and 3.6 μm in *P. cembra* and between 2.5 and 3.9 μm in *P. abies. Larix decidua* had a more pronounced variation between earlywood and latewood, as LRD and LA values decreased markedly from 42.9 to 2.8 μm and from 1228.5 to 30.7 μm^2^, respectively. Also, WT values increased sharply across the tree ring from 2.7 up to 7.6 μm over the first 75% of the ring and then decreased to ca. 5 μm in latewood (Figure 3, third column). A similar pattern was found in *P. mariana*, with LRD ranging between 35.2 and 3.3 μm and LA between 1056.4 and 60.3 μm^2^. The increase in WT across the tree ring, from 2.5 to 4.9 μm, also occurred in the first 75% of the ring. WT then decreased to 3.6 μm in the last part of latewood (Figure 3, fourth column).

All simulated anatomical features across the ring were in good agreement with the measured data for the studied species, with *R*^2^ varying between 0.86 and 0.99 (Figure 3). Divergences were noted between the observed and simulated values for the first and last cells. The calculated latewood percentage in both observed and simulated tree rings also showed small differences (Table 2), ranging from 0.5% for *P. abies* to 2.9% for *P. mariana*. Latewood percentage was underestimated in *P. abies* and overestimated in *L. decidua* and *P. mariana*.

**Table 2:** Observed and simulated percentages of latewood in the tree rings of different conifer species.

**Species**	**Observed (%)**	**Simulated (%)**
*Pinus cembra*	0	0
*Picea abies*	1.4	0.9
*Larix decidua*	55.6	57.8
*Picea mariana*	26.5	29.4

*Latewood was calculated using Mork's formula (Denne, 1989)*.

**Figure 4:**
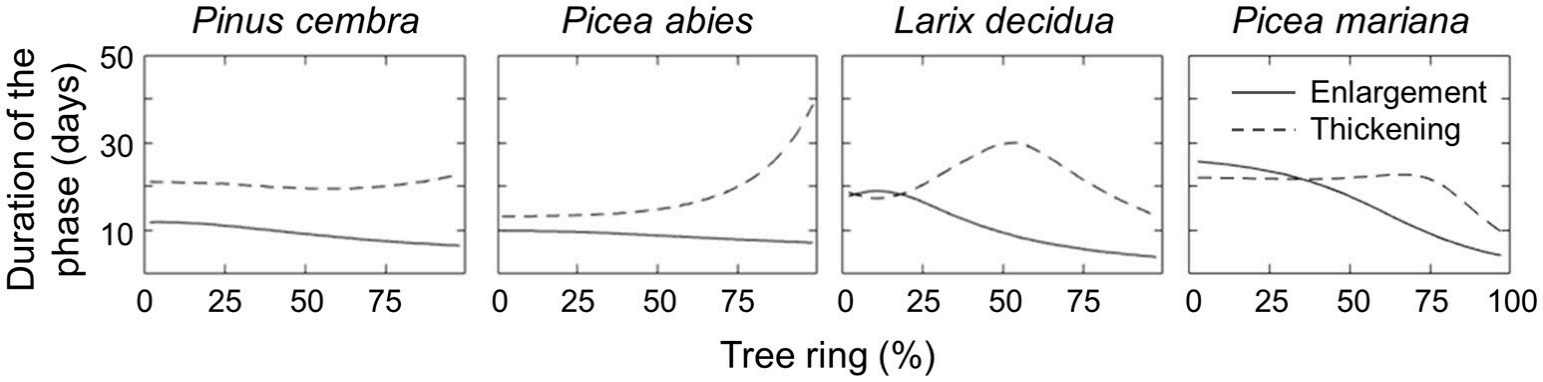
Estimated duration (days) of cell enlargement and cell-wall thickening for the four conifer species.

The model also estimated the duration of the enlargement and wall-thickening phases (Figure 4). The duration of enlargement (i.e., when cell area is increasing) decreased continuously across the tree ring for all the species (Figure 4, continuous lines). Smaller variations in the duration of enlargement were observed in *P. cembra* (from 11.7 to 6.5 days) and *P. abies* (from 9.9 to 7.2 days) with a constant decrease across the tree ring. A wider variation in enlargement duration was observed in *L. decidua* (from 19 to 3.9 days) and *P. mariana* (from 25.7 to 4.3 days) with a phase of pronounced decrease. However, the predicted duration of exhibited greater interspecific variability (Figure 4, dashed lines). *P. cembra* had an almost constant duration for cell-wall thickening across the ring with a slight increase in the last part of latewood (ranging from 20 to 23.7 days), while *P. abies* showed a pronounced increase in the duration of wall thickening for ca. 75% of the tree ring with values increasing from 13.2 to 39 days. In contrast, the simulations for *L. decidua* predicted an initial increase in the duration of the thickening phase over the first half of the tree ring (from 18.7 to 30 days), then followed by a decrease (down to 13.5 days). A similar pattern emerged for *P. mariana*, where the duration of the phase increased slightly over the first 75% of the ring (from 22 to 24 days) with a decrease to 9.7 days toward the end of the tree ring.

## Discussion

**Figure 5:**
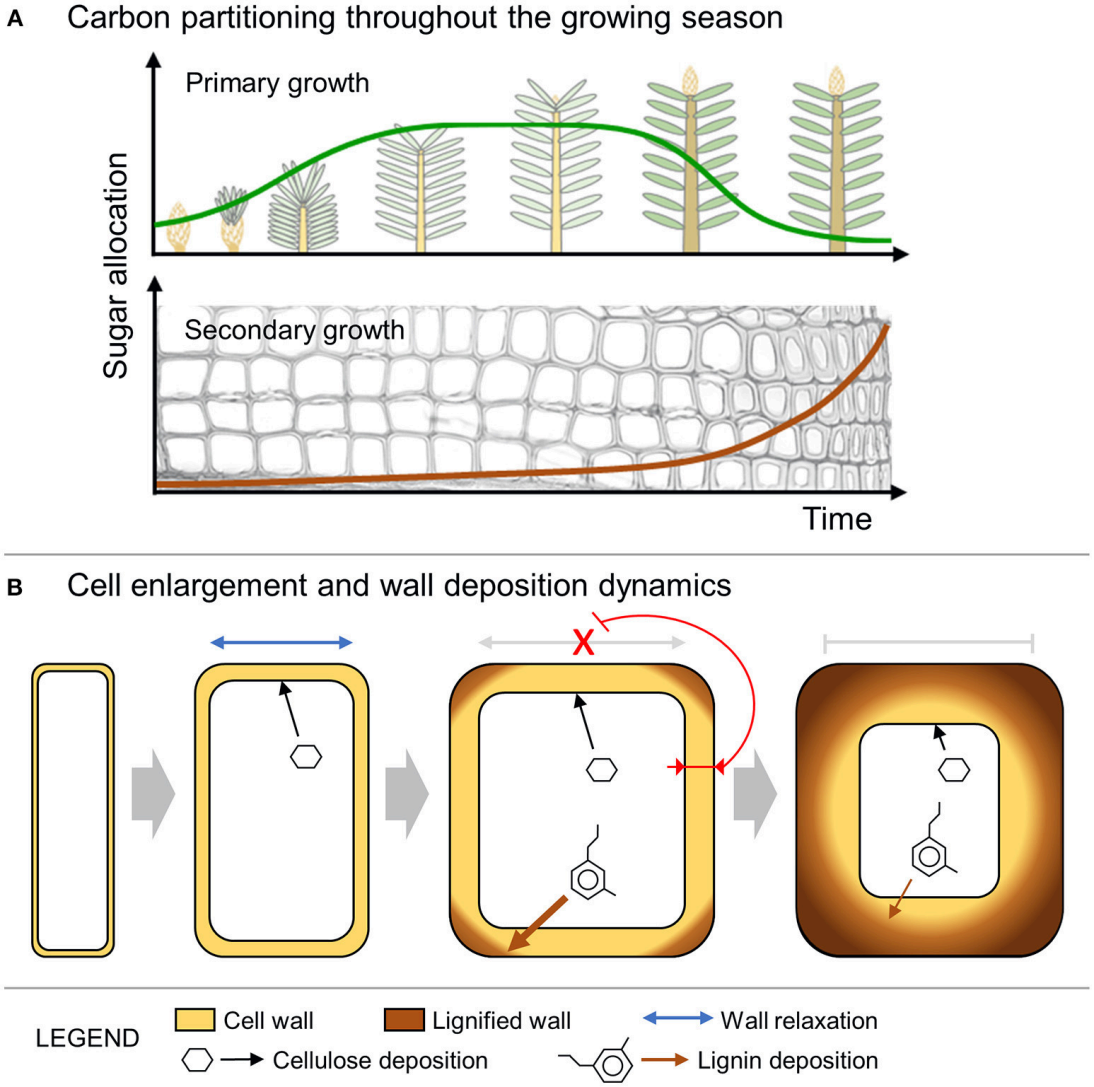
Schematic representation of the model dynamics. **(A)** Sugar allocation to either primary or secondary growth during the growing season. **(B)** Cell enlargement including wall relaxation and deposition of new cellulose. The expansion process stops when the increased thickness of the wall inhibits further relaxation. The wall thickening and lignification continue at a rate that is inversely proportional to the cell lumen perimeter (i.e., contact surface between the cytosol and the wall).

This paper presents a simulation model of tracheid development that assumes changes in tracheid anatomy from earlywood to latewood are coupled to the end of primary growth, when new shoots become sources of assimilates (Richardson and Dinwoodie, 1960; Gordon and Larson, 1968). The simulated tree-ring patterns were in good agreement with data collected from the four conifer species (Figure 3), supporting a causal link between changes in sugar availability and wood cell traits (Figure 5A).

A large amount of sugar is required for tree-ring development (Kagawa et al., 2006; Deslauriers et al., 2009, 2016; Klein et al., 2016) even though carbohydrate allocation to cambium has a lower ranking priority when compared to new foliage production (Minchin and Lacointe, 2005). This reasoning is based mainly on carbon allocation among different tree parts depending on the competitive ability of the sinks and their relative proximity to the carbon sources (Allen et al., 2005; Brüggemann et al., 2011); this influences the quantity allocated for wood formation.

The involvement of sugar availability on the development of tracheid traits is further reinforced by studies of carbon partitioning that demonstrate that the allocation of carbon lower down the stem decreased when shoots and needles were actively growing (Kagawa et al., 2006; Heinrich et al., 2015). Recently, Heinrich et al. (2015) found a downstream gradient of C allocation during canopy growth (15 days after bud break), meaning that sugar availability for stem growth decreases with tree height during earlywood formation. By studying tree-ring formation along a 9-m-tall *Picea abies*, Anfodillo et al. (2012) found that cell expansion in earlywood was less at the tree top—forming cells having smaller lumen—than at the tree bottom where cells had larger lumen; this explained the tapering pattern of the tree. Our model may also explain the increase in the diameter of vascular conduits toward the stem base (i.e., the tapering pattern of the conducting system) (Anfodillo et al., 2006). Therefore, the woody cell patterns both across the tree ring and along the hydraulic pathway could both be determined by C availability during the growing season and along the tree height, respectively.

In agreement with our model, several other studies provide interesting clues regarding sugar availability and the resulting anatomy in conifers. A phloem-girdling experiment performed several times during the growing season led to significant modifications in tracheid differentiation above the girdling (Winkler and Oberhuber, 2017). When phloem girdling was performed during earlywood formation, the newly formed tracheids had thicker walls and smaller lumen diameters; this agrees with our simulated earlywood-latewood cell development (Figure 2). Indeed, the authors explained that the girdling blocked carbon flow downwards, greatly increasing the availability of C above the girdling zone to produce the latewood-like cells. However, during latewood formation tracheids having larger lumen and thinner cell walls were produced, thus having characteristics more similar to earlywood cells. Nonetheless, the possible causes, such as wound response, were not well understood (Winkler and Oberhuber, 2017) and could not be explained by our model. Another convincing piece of evidence comes from detailed radial measurements of sugars and sucrose-metabolizing enzymes performed in the regions of cambial and differentiating cells at various times during the growing season (Uggla et al., 2001). While no differences in sugar concentrations were found between earlywood and latewood regions, an increased activity of sucrose-synthase (SuSy) was observed during the development of latewood tracheids (Uggla et al., 2001). SuSy is one of the main sucrose-cleaving enzymes and may reflect the greater incorporation of sugar during latewood formation due to increased biosynthesis of cell-wall components, such as polysaccharides (McFarlane et al., 2014) and lignin (Zhao and Dixon, 2011; Gerber et al., 2014).

Although the anatomical characteristics of wood encode environmental signals (Fonti et al., 2010; Fonti and Jansen, 2012)—as demonstrated by the clear mark left by light rings (Wang et al., 2000) or intra-annual density fluctuations (De Micco et al., 2016)—the processes involved remain tightly coupled to the physiological state of the tree (Balducci et al., 2016), and disentangling the direct and indirect effects remains a challenge (Battipaglia et al., 2016). Recently, Cuny and Rathgeber (2016) have shown a strong and positive effect of temperature on the wall deposition rate of the last latewood cells, but not in earlywood or transition wood cells. There was no observed climatic influence on the kinetics of cell enlargement and the resulting cell size. The lack of a clear climatic determinism to explain the decreasing tracheid diameter across the tree ring shows that the interactions between intrinsic and extrinsic factors are complex and likely species-specific. Furthermore, in the absence of major stresses, such as drought, the differentiation of tracheids is predominantly controlled by internal factors (Björklund et al., 2017; Rathgeber, 2017). Such observations are in good agreement with our model assumptions. Species living in the same environment produce different patterns of earlywood-latewood transitions. Different xylem anatomies also underlie different timings of xylem differentiation, as observed for the simulated species. Even if our model did not explicitly consider any specific climatic factor, the indirect effects of the environment are implicitly included in the availability of sugars since photosynthesis and growth dynamics of the primary meristems are under the control of light, water and temperature. It is also important to note that, in some cases, our simulations failed to correctly reproduce the traits of the last latewood cells (Figure 3). This can probably be explained by the fact that no direct effect of temperature was included in the model.

The duration of radial enlargement in cells, as estimated by the model, corresponds with that reported in conifers, which on average range between 7 and 35 days depending on the species, and this duration decreases moving across the tree ring (Wodzicki, 1971; Horacek et al., 1999; Rossi et al., 2006b). Fritts et al. (1999) proposed that cell radial growth depends more on the development rate, especially in spring, when the cell expansion rate is higher. However, our results suggest a greater importance of the duration of the enlarging process on cell size. This confirms the estimates of Cuny et al. (2013), in which the duration of enlargement contributed to 75% of the total change in cell diameter. Duration of cell-wall formation is generally longer than enlargement, lasting from 20–50 days, as it is more demanding in terms of resources. In general, secondary wall formation is reported to be shorter for cells located at the beginning of the tree ring longer for latewood cells, and decreasing again for the last latewood cells (Wodzicki, 1971; Rossi et al., 2006b; Cuny, 2013). The estimates of our model varied among the simulated species and corresponded only partially to the timings reported in the literature. This could be explained by the different species analyzed in this study, or by the greater importance of the cell-wall formation rate, with respect to duration, on the changes in wall radial thickness (Cuny, 2013).

For more than 45 years, the role of auxin in plant cell growth has been based on the acid-growth theory to explain the auxin-dependant alteration of cell-wall rigidity that enables wall loosening and thus cell growth (Rayle and Cleland, 1970, 1992; Dünser and Kleine-Vehn, 2015). Recently, novel insights have revealed that auxin provokes not only cell-wall acidification and loosening (promoting growth) but also vacuole morphogenesis (promoting or limiting growth). Through auxin-mediated actions and water intake, the vacuole has an important space-filling function during cell growth (Scheuring et al., 2016) that efficiently controls growth independent of the wall and neighboring cells (Dünser and Kleine-Vehn, 2015). However, larger vacuoles do not necessarily increase cell size as the rigidity of the cell wall ultimately restricts cellular enlargement (Löfke et al., 2015). Thus, to these important auxin-mediated mechanisms occurring during the primary cell wall growth, our model adds important feedbacks to the secondary cell-wall deposition processes.

From a biomechanical point of view, it is assumed that cell enlargement is limited by thicker cell walls (Hamant and Traas, 2010; Huang et al., 2012). Furthermore, cell enlargement could also be constrained by the synthesis and deposition of some secondary wall layers (S1 or even S2 layers), which occur concomitantly with lignification spreading from the middle lamella and the corners of the primary wall (Donaldson, 2001). In this work, we propose that cell enlargement is progressively slowed during the deposition of subsequent layers of cell wall because of the inhibition of wall relaxation processes and the decreasing accessibility of polysaccharides and pectins to the outer wall layers near the middle lamella (Figure 5B). With the progressive deposition of new wall layers, the possibility for cell-wall relaxation and cell growth is increasingly inhibited and in our model is blocked at a threshold value of the cell-wall thickness (WT^*^). After the cessation of cell enlargement, several layers of secondary wall are then deposited and progressively lignified. The biosynthesis processes of cellulose and non-cellulosic polysaccharides are known to be tightly coupled with the biosynthesis and polymerization of lignin (Roussel and Lim, 1995; Ruel et al., 2006). Both cell-wall deposition and lignification rates are likely differently influenced by the contact surface between the cell wall and the lumen (i.e., lumen perimeter).

In this study, we built a process-based model able to simulate the intra-annual variation of the anatomical traits of tracheids in four different conifer species that result from the seasonal changes in available sugar. The main difference between our model and previous models is that the processes of cell enlargement and wall deposition are not separated in time and space, i.e., the two processes occur at the same time and interact with each other. In particular, we assumed that cell enlargement is regulated by the deposition of successive layers of secondary wall that induce the stiffening of the wall and the cessation of cell expansion (Dünser and Kleine-Vehn, 2015). All other models of xylogenesis assume that differentiating cells can either expand or form secondary walls. Different assumptions have been made for the transition from one phase to the other. Most models simply assume that the end of the enlargement phase signals the beginning of the wall deposition phase (e.g., Fritts et al., 1999; Hölttä et al., 2010; Vaganov et al., 2011; Drew and Downes, 2015; Li et al., 2017) and very few models provide details of the processes that regulate this transition. Hartmann et al. (2017) developed a spatially explicit model considering a morphogen gradient (e.g., auxin) defining the boundary between the enlargement and cell-wall–thickening zones and tested if its change during the season could be responsible for the known changes in cell traits across the tree rings. Our model did not explicitly consider spatial dynamics (e.g., diffusion of sugars and other morphogenetic signals) nor cambial cell division, which influences the position of the developing tracheids from the main source of nutrients (i.e., the phloem). In fact, the model assumed that each cell grows independently of each other (as also proposed by Dünser and Kleine-Vehn, 2015), showing how spatial gradients are not strictly necessary for the emergence of the typical tree-ring patterns observed in conifers. However, inclusion of cell division and cell–cell interactions (i.e., spatial dynamics) could improve the predicted timings of cell enlargement and secondary wall deposition.

The presented results highlight the usefulness of focusing on the relation between basic cellular processes (e.g., carbon metabolism, cell expansion and wall deposition) and emergent properties at the tissue-scale (e.g., xylogenesis). In particular, the model has the potential to link such processes with the effects of climatic factors, mainly temperature and water, to investigate the variability of wood density profiles and the appearance of intra-annual density fluctuations in Mediterranean environments.

## Author contributions

FC, AD, SR and FG planned and designed the research under the coordination of HM and SM. All authors contributed jointly to model development. FG and FC carried out the mathematical implementation of the model. FC and AD wrote most of the manuscript with substantial contributions from VD, SR and SM.

### Conflict of interest statement

The authors declare that the research was conducted in the absence of any commercial or financial relationships that could be construed as a potential conflict of interest.
